# Editorial: Research on nanomaterials in tumor diagnosis and therapy, volume II

**DOI:** 10.3389/fbioe.2026.1829708

**Published:** 2026-03-25

**Authors:** Zihao Yan, Peng Liu, Yue Kang, Huizhe Xu, Shenglong Li

**Affiliations:** 1 Department of Neurosurgery, Cancer Hospital of Dalian University of Technology, Liaoning Cancer Hospital and Institute, Shenyang, Liaoning, China; 2 Department of Electromagnetic Compatibility, Liaoning Medical Device Testing Institute, Shenyang, Liaoning, China; 3 Cancer Hospital of Dalian University of Technology, Cancer Hospital of China Medical University, Liaoning Cancer Hospital and Institute, Shenyang, Liaoning, China; 4 Central Laboratory, Cancer Hospital of Dalian University of Technology, Liaoning Cancer Hospital and Institute, Shenyang, Liaoning, China; 5 Second Ward of Bone and Soft Tissue Tumor Surgery, Cancer Hospital of Dalian University of Technology, Cancer Hospital of China Medical University, Liaoning Cancer Hospital and Institute, Shenyang, Liaoning, China

**Keywords:** cancer, nanomaterials, nanomedicine, therapy, tumor diagnosis

The integration of Biomedical Engineering (BME) with broader multidisciplinary collaboration, is increasingly recognized as an essential direction for future medical research and clinical diagnosis and treatment (Ghasemlou et al., 2024; Tavakkoli Yaraki et al., 2022). In recent years, investigations on biomedical nanomaterials have flourished, with tumor-related nanomaterials accounting for the largest proportion of these studies. As the field has progressed, the focus has gradually shifted from the intrinsic properties and targeted delivery capabilities of nanomaterials to their roles in modulating the tumor microenvironment (TME) and regulating tumor immunity and metabolism (Kang et al., 2023; Jain et al., 2026). This Research Topic brings together the latest research findings and viewpoints in the field of nanomaterials in tumor diagnosis and therapy, with a particular focus on the interplay between nanomaterials and tumor immune regulation. The content mainly covers three aspects: enhancing antitumor immunity by remodeling the tumor immune microenvironment (TIME) (Li D. et al., 2026), synergizing with immune checkpoint therapies (De Leon et al., 2026), and overcoming tumor drug resistance while further promoting immune activation-mediated by nanomaterials (Khan et al., 2026).

Regarding the remodeling of the tumor microenvironment by nanomaterials, Piao et al. provided a comprehensive overview of the TME and tumor immunotherapy. They summarized how various nanomaterials regulate the TIME, spanning innate immune activation, adaptive immune responses, and the reversal of tumor-induced immunosuppression. The study clearly delineates the mechanisms by which each class of nanomaterials contributes to tumor immunotherapy, with particular emphasis on the potential of hybrid nanomaterials in modulating the TIME. The authors also proposed a forward-looking concept of establishing standardized nanomaterial-based therapeutic strategies guided by tumor immune networks. Mechanistically, Li et al. systematically described the unique advantages of nanomaterials over conventional drugs in overcoming the complex pH and physicochemical environment of the digestive tract to achieve targeted drug delivery to tumors. The authors pointed out that these formulations can activate immunity in digestive tract tumors, promote adaptive antitumor immune responses, enhance CD8^+^ T-cell infiltration, and reverse M2 macrophage polarization, thereby reshaping the immunosuppressive milieu. Furthermore, they proposed an advanced concept of selecting personalized delivery strategies tailored to tumor heterogeneity. Yan et al. similarly focused on the role of nanomaterial-based delivery systems in modulating the TME. By employing bacteria as carriers, these systems can not only selectively target to the tumor core but also be assembled with nanomaterials to achieve efficient drug delivery. Moreover, the intrinsic antigenic properties of bacteria can serve as adjuvants to activate adaptive immunity, offering a novel perspective for cancer immunotherapy (Zhang J. et al., 2026). Gao et al. summarized the critical roles of cancer-associated fibroblasts (CAFs) in multiple tumor behaviors, including extracellular matrix remodeling, immune cell infiltration, metabolism, and tumor invasion and metastasis. CAFs are closely associated with tissue stiffness, an emerging focus in cancer research, and remodeling the overall TME may more effectively modulate tissue stiffness than conventional targeted therapy. The development of nanomaterials that selectively target specific CAF subtypes in combination with immunotherapy is likely to be a key direction for future investigation (Chen et al., 2026). Clinically, Cai et al. conducted a multicenter, large-cohort retrospective study in triple-negative breast cancer (TNBC) and found that pegylated liposomal doxorubicin (PLD) exhibited lower toxicity and milder side effects compared with conventional doxorubicin. They also identified that the proportion of stromal tumor-infiltrating lymphocytes (sTILs) serves as an independent prognostic factor in TNBC, highlighting the critical role of the immune landscape and immunotherapy in the management of breast cancer.

Chen et al. focused directly on the synergistic use of nanomaterials and immune checkpoint inhibitors. In their perspective, they outlined the potential advantages of such combination therapies as well as the key challenges that remain, providing guidance for future research. Xue et al. developed dual immune checkpoint receptor-coated nanovesicles that block the ligands PD-L1 and Galectin-9, thereby activating T cells and eliciting antitumor immune responses. These nanovesicles were shown to convert the TME into a ‘hot’ state, achieving significant tumor suppression without notable organ toxicity, thus laying the foundation for the development of more precise and effective immunotherapies.

Guan et al. reviewed the various mechanisms by which nanomaterials can overcome therapeutic resistance in breast cancer. In addition, through their photothermal and magnetic heating effects, as well as the ability to target specific signaling pathways, nanomaterials can indirectly activate antitumor immunity. Combining nanomaterials with immunotherapy may produce synergistic effects exceeding the sum of their individual actions. Xu et al. highlighted the pivotal role of nanomaterials in overcoming therapeutic resistance in liver cancer. They systematically reviewed nanomaterial-based studies and applications for three commonly used targeted therapies and noted that combining nanomaterials with immune checkpoint inhibitors represents a promising therapeutic strategy. This synergistic effect is corroborated by photothermal and photodynamic therapies in liver cancer, which remodel the TME and activate immunity.

In summary, with deeper insights into tumor biology and rapid advances across multiple disciplines, cancer therapy has evolved from single-target strategies to multimodal combination therapies (Qi et al., 2026). Based on the findings and perspectives compiled within this Research Topic, it is evident that dynamic processes such as immune activation and metabolic regulation in tumors are emerging as core targets for the development of antitumor nanomaterials. Notably, immune and metabolic regulation in tumors does not involve a single or limited set of pathways. Moving forward, we propose that researchers design antitumor nanomaterials with dynamic regulatory capabilities based on the tumor immune and metabolic networks (Liu et al., 2026; Zhang B. et al., 2026; Li W. et al., 2026). It is well recognized that the TME exhibits substantial intratumoral and interpatient heterogeneity (Chapman et al., 2023). Accordingly, the development of antitumor nanomaterials should be adapted to the immune landscape of each tumor for personalized precision therapy. Finally, to elicit a comprehensive antitumor immune response, tumor evolution and immune evasion mechanisms also warrant attention (Spitzer et al., 2025). Designing antitumor nanomaterials that target tumor clones and their clone neoantigens may represent a promising avenue for future exploration (Xia et al., 2024; Luo et al., 2025; Zhou et al., 2025).

## References

[B1] Chapman O. S. Luebeck J. Sridhar S. Wong I. T. Dixit D. Wang S. (2023). Circular extrachromosomal DNA promotes tumor heterogeneity in high-risk medulloblastoma. Nat. Genetics 55 (12), 2189–2199. 10.1038/s41588-023-01551-3 37945900 PMC10703696

[B2] Chen Z. Y. Liu H. Z. Shang Z. J. Luo G. F. Zhang X. Z. (2026). Nanomedicine for targeting cancer-associated fibroblasts in cancer therapy. Theranostics 16 (3), 1545–1576. 10.7150/thno.120283 41355964 PMC12679702

[B3] De Leon G. Zhang L. Siddiqui N. A. Naguib N. Chen F. Padmanabhan R. (2026). An ultrasmall core-shell silica nanoparticle improves antitumour immunity and survival by remodelling suppressive melanoma microenvironments. Nat. Nanotechnology 21 (2), 311–322. 10.1038/s41565-025-02083-z 41461940 PMC12916478

[B4] Ghasemlou M. Pn N. Alexander K. Zavabeti A. Sherrell P. C. Ivanova E. P. (2024). Fluorescent nanocarbons: from synthesis and structure to cancer imaging and therapy. Adv. Materials Deerf. Beach, Fla 36 (19), e2312474. 10.1002/adma.202312474 38252677

[B5] Jain M. Est-Witte S. E. Shannon S. R. Neshat S. Y. Yu X. Dunham S. (2026). Biodegradable targeted polymeric mRNA nanoparticles enable *in vivo* CD19 CAR T cell generation and lead to B cell depletion. Sci. Advances 12 (11), eadz1722. 10.1126/sciadv.adz1722 41811961 PMC12978253

[B15] Kang N. Zhang S. Wang Y. (2023). A personalized mRNA vaccine has exhibited potential in the treatment of pancreatic cancer. Holist. Integr. Oncol. 2 (1), 18. 37323470 10.1007/s44178-023-00042-z PMC10248956

[B6] Khan M. S. Alqahtani T. Al Shmrany H. Gupta G. Goh K. W. Sahebkar A. (2026). Enhanced permeability and retention (EPR) effect: advances in nanomedicine for improved tumor targeting. Biomater. Advances 181, 214636. 10.1016/j.bioadv.2025.214636 41365275

[B7] Li D. Cui G. Yang K. Lu C. Jiang Y. Zhang L. (2026a). Inhibiting macrophage-derived lactate transport restores cGAS-STING signalling and enhances antitumour immunity in glioblastoma. Nat. Cell Biology 28 (2), 349–362. 10.1038/s41556-025-01839-y 41495200

[B8] Li W. Tang Z. Xue J. (2026b). Synergistic STING activation and oxidative cascades-induced ferroptosis drive tumor microenvironment remodeling by engineered manganese nanoreactors. Redox Biology 89, 103977. 10.1016/j.redox.2025.103977 41496203 PMC12808843

[B9] Liu Y. Sun D. Li W. Zhang Z. Li C. Che X. (2026). Nanomedicine-mediated modulation of tumor metabolism for enhanced immunotherapy. J. Controlled Release Official Journal Control. Release Soc. 390, 114587. 10.1016/j.jconrel.2025.114587 41478378

[B10] Luo J. Q. Zhang J. Yu H. H. Li Y. X. Zhou Z. H. Duan Y. (2025). Genetically engineered biomimetic nanoparticles for synergistic activation of glioma-associated macrophages against glioblastoma. J. Am. Chem. Soc. 147 (41), 37641–37657. 10.1021/jacs.5c12775 41042815

[B11] Qi B. Xiao Z. Zhu Y. Tang S. Zhao Z. Chen G. (2026). Self-disassembling diatomic nanocluster bomb unlock reciprocal synergistic multi-pathway cancer therapy. J. Nanobiotechnology. 10.1186/s12951-026-04245-0 41814299 PMC13094113

[B12] Spitzer A. Johnson K. C. Nomura M. Garofano L. Nehar-Belaid D. Darnell N. G. (2025). Deciphering the longitudinal trajectories of glioblastoma ecosystems by integrative single-cell genomics. Nat. Genetics 57 (5), 1168–1178. 10.1038/s41588-025-02168-4 40346362 PMC12081298

[B13] Tavakkoli Yaraki M. Liu B. Tan Y. N. (2022). Emerging strategies in enhancing singlet oxygen generation of nano-photosensitizers toward advanced phototherapy. Nano-micro Letters 14 (1), 123. 10.1007/s40820-022-00856-y 35513555 PMC9072609

[B14] Xia Z. Jin Q. Long Z. He Y. Liu F. Sun C. (2024). Targeting overexpressed antigens in glioblastoma via CAR T cells with computationally designed high-affinity protein binders. Nat. Biomedical Engineering 8 (12), 1634–1650. 10.1038/s41551-024-01258-8 39420062

[B16] Zhang J. Shao T. Qu F. Bai C. Li S. Gao P. (2026). Bacterial outer membrane vesicles in potentiating cancer vaccines: progress and prospects. Adv. Science Weinheim, Baden-Wurttemberg, Ger., e21684. 10.1002/advs.202521684 41709766 PMC13205634

[B17] Zhang B. Zhao L. Li H. Wang N. Wang X. Shang L. (2026). Glycolysis in the tumor microenvironment shapes dendritic cell function and antitumor immunity. Front. Immunology 17, 1744671. 10.3389/fimmu.2026.1744671 41737218 PMC12926433

[B18] Zhou S. Wang C. Ning Y. Wang Y. Xin F. Ren L. (2025). Virus-like particle-based personalized neoantigen nano-vaccine for tumor immunotherapy and recurrence prevention. ACS Nano 19 (40), 35385–35400. 10.1021/acsnano.5c06278 41032465

